# Allosteric Modulation of Adenosine A_2A_ Receptors as a New Therapeutic Avenue

**DOI:** 10.3390/ijms23042101

**Published:** 2022-02-14

**Authors:** Mustafa Korkutata, Lokesh Agrawal, Michael Lazarus

**Affiliations:** 1 Department of Neurology, Division of Sleep Medicine, Beth Israel Deaconess Medical Center, Harvard Medical School, Boston, MA 02215, USA; mkorkuta@bidmc.harvard.edu; 2 Molecular Neuroscience Unit, Okinawa Institute of Science and Technology Graduate University, Kunigami-gun, Onna 904-0412, Japan; lokesh.agrawal@oist.jp; 3 International Institute for Integrative Sleep Medicine (WPI-IIIS), University of Tsukuba, Tsukuba 305-8575, Japan

**Keywords:** adenosine A_2A_ receptors, allosteric modulator, insomnia, slow-wave sleep, inflammation, cardiovascular function, body temperature, drug development

## Abstract

The therapeutic potential of targeting adenosine A_2A_ receptors (A_2A_Rs) is immense due to their broad expression in the body and central nervous system. The role of A_2A_Rs in cardiovascular function, inflammation, sleep/wake behaviors, cognition, and other primary nervous system functions has been extensively studied. Numerous A_2A_R agonist and antagonist molecules are reported, many of which are currently in clinical trials or have already been approved for treatment. Allosteric modulators can selectively elicit a physiologic response only where and when the orthosteric ligand is released, which reduces the risk of an adverse effect resulting from A_2A_R activation. Thus, these allosteric modulators have a potential therapeutic advantage over classical agonist and antagonist molecules. This review focuses on the recent developments regarding allosteric A_2A_R modulation, which is a promising area for future pharmaceutical research because the list of existing allosteric A_2A_R modulators and their physiologic effects is still short.

## 1. Introduction

Adenosine is a naturally occurring purine nucleoside that regulates various physiologic functions, including inflammation and wound healing, cardiac contraction, blood vessel formation, vasodilation, learning, memory, sleep, and arousal [1,2,3,4,5,6,7]. Adenosine is released by neurons and glial cells [8]. Extracellular adenosine modulates neuronal excitability, synaptic plasticity, and the release and reuptake of several neurotransmitters [9,10,11,12]. The effects of extracellular adenosine are modulated via four subtypes of G-protein coupled adenosine receptors (GPCRs), denoted A_1_, A_2A_, A_2B_, and A_3_ [13]. Adenosine A_2A_ receptors (A_2A_Rs) are broadly expressed in the brain, cardiovascular system, blood vessels, spleen, thymus, leukocytes, and lung, making them an important drug target [14]. This review focuses on allosteric A_2A_R modulation and the latest developments in this emerging field.

The therapeutic potential of targeting A_2A_Rs has prompted the development of numerous antagonist and agonist molecules to selectively control A_2A_R function. The myriad A_2A_R agonists and antagonists are considered potential therapeutic agents for inflammation, sickle cell disease, ischemia-reperfusion injury, and central nervous system (CNS) diseases [15,16]. The A_2A_R agonist regadenoson was approved by the US Food and Drug Administration to boost blood flow during cardiac stress tests [16]. Many other agonists and antagonists are undergoing clinical trials.

Medicinal chemists have made many efforts to develop small molecules as allosteric modulators in recent years. Unlike agonist and antagonist molecules, allosteric modulators evoke a selective physiologic response only where and when the orthosteric ligand is released [17]. GPCRs, including adenosine receptors, are allosterically regulated [17,18]. The list of existing allosteric A_2A_R modulators is short, however, and the physiologic opportunities for modulators are just emerging, making allosteric A_2A_R modulation a promising area for future research.

## 2. Adenosine and Its Receptors

Adenosine was initially recognized as a physiologic regulator of coronary vascular tone; since then, a growing body of reports indicates that adenosine regulates cellular functions through specific receptors present on the cell surface [19,20,21]. Adenosine is an endogenous purine nucleoside consisting of adenine and *D*-ribose, and is formed through hydrolysis of *S*-adenosylhomocysteine or adenosine monophosphate [22,23]. Adenosine formation from *S*-adenosylhomocysteine relies on the intracellular activity of the enzyme *S*-adenosylhomocysteine hydrolase, which bi-directionally assures the constant occupancy of a bound adenosine concentration in the cells [24]. Different enzymes mediate the formation of adenosine from adenosine monophosphate at both intracellular and extracellular levels.

Although adenosine does not exclusively act on synapses and is not stored in synaptic vesicles, it has a direct role in synaptic processes and the regulation of various neurotransmitters in the CNS. Nucleoside transporters mediate adenosine release and reuptake mechanisms through a concentration gradient between the intracellular and extracellular spaces. Therefore, adenosine is postulated as a modulator that affects neurotransmitter release and neuronal hyper- or depolarization and regulates glial cells [25]. Despite the modulatory role of adenosine, neurotransmitter properties are also observed for adenosine, which is due to the presence of the adenosine-producing enzyme in synapses. Extracellular adenosine acts on neurons through specific adenosine receptors [26].

Purinergic receptors are the natural target of purine molecules such as adenosine and adenosine triphosphate. These receptors were recognized for the first time in 1978 [27]. Two types of purinergic receptors, P1 and P2, were subsequently identified based on their pharmacologic profile [28]. P1 receptors recognize adenosine as a primary natural ligand and are therefore also called adenosine receptors. Each of the four types of adenosine receptors, A_1_R, A_2A_R, A_2B_R, or A_3_R, is characterized by a distinct pharmacologic profile. These receptors are members of the GPCR superfamily [17]. A_2A_Rs and A_2B_Rs are Gs-coupled receptors, and their activation increases the activity of adenylyl cyclase, the enzyme that initiates cyclic AMP (cAMP) synthesis in the cells. A_1_Rs and A_3_Rs are Gi/q coupled receptors, and their activation through adenosine or agonist molecules inhibits the activity of adenylyl cyclase, which suppresses cAMP synthesis in the cells.

## 3. A_2A_R and Its Physiologic Roles

The four types of adenosine receptors, A_1_R, A_2A_R, A_2B_R, or A_3_R, react with extracellular adenosine [13]. The activation of A_2B_Rs reportedly requires a high adenosine concentration. Unlike A_2B_Rs, adenosine levels under basal physiologic conditions are adequate to activate A_1_Rs, A_2A_Rs, and A_3_Rs with relatively equal potency. The pharmacologic strength of an endogenous ligand or agonist at its receptor, however, relies on the number of receptors on the cells. Higher concentrations of adenosine are needed to show an effect in the presence of only a few receptors. Local expression of the A_1_Rs and A_2A_Rs in the brain is suggested to be relatively higher than that of the other two adenosine receptors [6,29].

**Figure 1:**
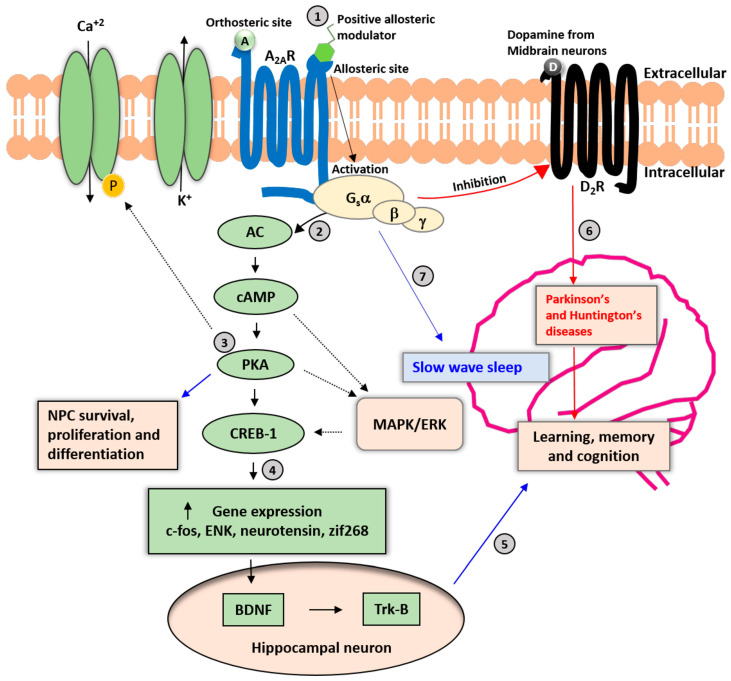
Neuronal A_2A_R signaling cascades. A_2A_R is a Gs(olf)-protein-coupled receptor involved in various physiologic processes. (1) The allosteric modulation sites may be pharmacologically relevant for avoiding adverse effects on the cardiovascular and other peripheral systems. (2) Binding of adenosine and an allosteric modulator to A_2A_Rs enhances the activation of cyclic adenosine monophosphate (cAMP) and protein kinase A (PKA), resulting in the phosphorylation of calcium ion channels and increased influx of Ca^+2^ into the cytoplasm. (3) The PKA pathway also promotes neural progenitor cell (NPC) survival, proliferation, and differentiation; and activation of the mitogen-activated protein (MAP)-kinase pathway. (4) PKA-mediated phosphorylation of the cAMP-responsive element binding protein 1 (CREB-1) regulates the expression of genes such as c-fos, enkephalin (ENK), neurotensin, and zinc finger protein 268 (zif268). (5) The secretion of brain-derived neurotrophic factor (BDNF) and activation of tropomyosin receptor kinase B (TrkB) receptors in response to A_2A_R activation in hippocampal neurons may be relevant for cognitive functions such as learning and memory. (6) A_2A_R activation may be a counter mechanism to control the activation and expression of dopamine D_2_ receptors (D_2_Rs). Long-term imbalance of D_2_R signaling leads to impairments in cognitive and motor functions and the development of Parkinson’s and Huntington’s diseases. (7) Activation of A_2A_R in the nucleus accumbens increases slow-wave sleep in mice. Solid black arrows represent the primary signaling pathway of A_2A_Rs, and dashed black arrows represent secondary signaling pathways. A: Adenosine; D: Dopamine.

A_2A_Rs were first identified by Libert and colleagues when they cloned several orphan GPCRs from the dog thyroid [30]. Afterward, A_2A_Rs were cloned from other species, including guinea pigs, mice, rats, and humans [31,32,33,34]. As with the other GPCRs, A_2A_Rs induce classical secondary messenger pathways. The A_2A_R signaling pathway may vary depending on the cell and tissue type in which the receptors occur. For example, Gs is the major G-protein associated with A_2A_Rs in the peripheral system. On the other hand, A_2A_Rs in the striatum, where they are highly expressed, mediate their effects mainly through Golf activation in the rat. Active Gs and Golf proteins stimulate adenylyl cyclase (Figure 1) which increases cellular cAMP levels and activates protein kinase A (PKA) which then phosphorylates and promotes cAMP-responsive element-binding protein 1 (CREB1) [16,35]. Activation of A_2A_Rs also activates extracellular signal-regulated kinases (ERK) and several other kinases of the mitogen-activated protein kinase (MAPK) family which trigger specific cellular responses [36]. A_2A_Rs form heterodimer structures with other GPCRs (e.g., metabotropic glutamate type 5 receptor (mGluR5)/A_2A_R, cannabinoid receptor type 1 (CB_1_)/A_2A_R, dopamine D_2_ receptor (D_2_R)/A_2A_R, dopamine D_3_ receptor/A_2A_R), and even CB_1_/A_2A_R/D_2_R heterotrimers [37,38,39,40,41].

The development of electron microscopy, selective radioligands, and antibodies has greatly contributed to A_2A_R distribution mapping. Furthermore, advancements in electron microscopy helped to determine the interactions of agonists and antagonists with their receptors and receptor density in particular regions. A_2A_Rs are concentrated on GABAergic medium-sized spiny neurons of the striatum, core and shell regions of the nucleus accumbens (NAc), olfactory tubercule, and dopamine-rich areas of the brain [16].

**Figure 2:**
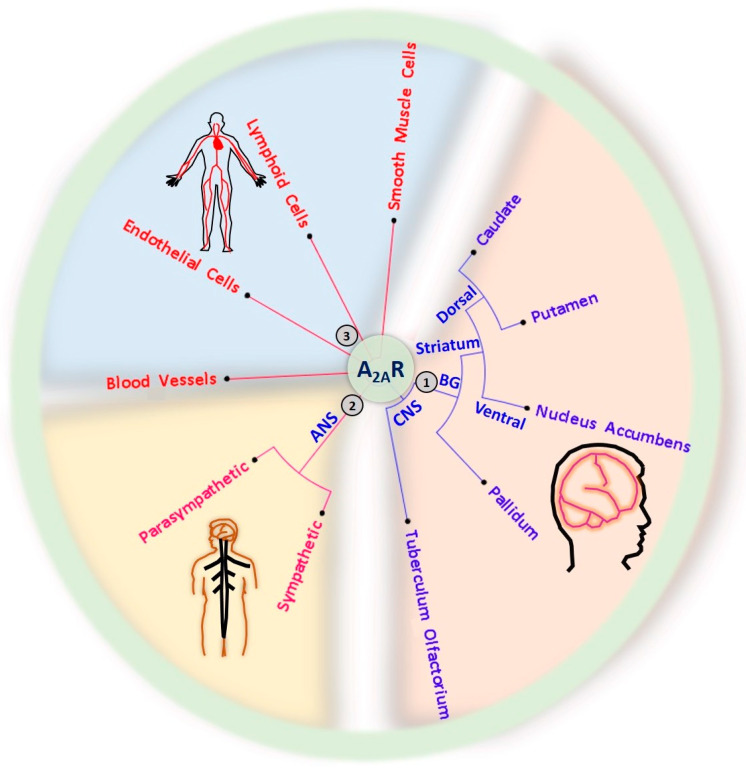
Expression of A_2A_Rs in the central nervous system (CNS), autonomic nervous system (ANS), circulatory system, and musculoskeletal system. (1) CNS A_2A_Rs are mainly expressed in the basal ganglia (BG), including the dorsal pallidum, the nucleus accumbens in the ventral part of the striatum, and the dorsal striatum comprising the caudate and putamen. (2) A_2A_Rs are also expressed in the sympathetic and parasympathetic ANS. (3) The distribution of A_2A_Rs is not limited to the nervous system; A_2A_Rs are also found in the circulation system, including heart, blood vessels, lymphoid cells (immune cells), and smooth muscle cells of the musculoskeletal system.

A_2A_Rs play a significant role in regulating the indirect pathways of the basal ganglia in the brain (Figure 2) [16]. The basal ganglia have an evolutionarily conserved essential role in learned habits, goal-directed movements, and locomotion [42]. The basal ganglia carry out their functions through direct and indirect circuits, originating in conspicuous populations of striatal medium spiny neurons that project to different output structures. Direct pathway neurons express excitatory dopamine D_1_ receptors (D_1_Rs) and inhibitory A_1_Rs, whereas indirect pathway neurons express inhibitory D_2_Rs and excitatory A_2A_Rs [43]. Studies in mice revealed that both direct- and indirect pathway medium spiny neurons are active during mouse locomotion but quiescent during inactive phases [44], and chemogenetic activation of direct and indirect pathways neurons increases and decreases locomotor activity, respectively [45]. Moreover, recent findings indicate that optogenetic activation of indirect pathway neurons in the NAc, a part of the brain that is associated with motivation and pleasure, induces slow-wave sleep, whereas inhibition suppresses slow-wave sleep [46]. Other observations show that when an action does not result in a reward, increased activity of indirect pathways occurs, suggesting a role of the indirect pathways in controlling goal-directed behavior [47].

Apart from medium-sized spiny neurons in the basal ganglia, A_2A_Rs are expressed in various other tissues, including smooth muscle cells, thymus, blood platelets, endothelial and lymphoid cells, leukocytes, spleen, blood vessels, lung, heart, and neurons in sympathetic and parasympathetic nervous systems [14,48] (Figure 2). A_2A_Rs have a wide range of physiologic functions in the body, such as protecting tissues from inflammatory damage, mediating vasodilation, and supporting the formation of new blood vessels.

Several A_2A_R agonists and antagonists are currently in clinical trials. The selective A_2A_R agonist regadenoson was approved by the US Food and Drug Administration to increase blood flow in cardiac nuclear stress tests. On the other hand, the effects of A_2A_R antagonists for the treatment of Parkinson’s disease (PD) are promising. Other trials have been conducted with several agonists and antagonists aimed at treating infectious disease, ischemia-reperfusion injury, cancer, inflammation, sickle cell disease, diabetic nephropathy, and other CNS disorders. The increasing number of reports and patents demonstrates the growing interest in targeting the A_2A_R [16].

## 4. The Concept of Allosteric Modulation

The most common method to stimulate receptors in pharmacology and biochemistry is to target orthosteric sites with their endogenous ligand, agonists, or antagonists. On the other hand, studies show that receptor activity can be altered by small molecules that bind to an allosteric site different from the site where the endogenous ligand, agonists, or antagonists would bind [49]. The small molecules that bind to the allosteric sites of the receptors are termed allosteric modulators. Unlike endogenous ligands, agonists, or antagonists, an allosteric modulator cannot itself activate or inactivate receptors but alters the receptor’s response to substrates that bind to orthosteric sites in two ways: (1) increase or decrease affinity, i.e., the ability of orthosteric substances to bind receptors, and (2) increase/decrease efficacy, i.e., the ability of orthosteric substances to activate receptors [50]. Allosteric modulators reportedly change the receptor conformation, which alters the effect of the endogenous ligand, agonist, and antagonist binding [51]. The concept of receptor modulation is not straightforward with respect to practical implementation. Allosteric modulators do not necessarily equally alter the affinity and efficacy of endogenous ligands, agonists, or antagonists of the receptors. An allosteric modulator may alter the efficacy or affinity of the endogenous ligand, but not that of the agonist or antagonist of the receptors or vice versa [52].

The term ‘allostery’ was first used in enzymology studies in the early 1960s [53,54,55]. Subsequently, allosteric modulation has been identified for all receptor superfamilies, including GPCRs, nuclear hormone receptors [56,57], receptor tyrosine kinases [58,59], and ligand/voltage-gated ion channels [60,61,62,63,64]. The term “allosteric” began to be used increasingly in the literature, and a broad spectrum of allosteric modulators was described. Consequently, the classification of allosteric modulators was necessary to avoid possible confusion [65,66,67]. Three properties are considered in the classification of allosteric modulators: (1) affinity modulation of the orthosteric ligand, (2) modulation of the signaling effect of the orthosteric ligand, and (3) direct effects of the allosteric modulator in the absence of the orthosteric ligand. Moreover, allosteric modulators are classified in terms of their effects on orthosteric ligands as positive allosteric modulators (PAM), negative allosteric modulators (NAM), or silent allosteric modulators, also known as neutral allosteric ligands [68]. PAMs enhance the agonist/antagonist affinity and efficacy, whereas NAMs decrease orthosteric ligand affinity and efficacy. Unlike PAMs and NAMs, silent allosteric modulators do not affect the agonist or antagonist activity of orthosteric ligands, but bind to the allosteric site of the receptors and prevent PAMs or NAMs from binding to the same site, thereby inhibiting the activity of positive/negative allosteric modulators [52]. It is important to note that activities of allosteric modulators are therefore limited by where and when the orthosteric ligand is released. Thus, in contrast to agonists or antagonists, allosteric modulators promise greater safety and fewer side effects in therapeutic applications.

## 5. Allosteric A_2A_R Modulation

Adenosine receptors are among the first known allosterically regulated GPCRs. Early studies demonstrated that amiloride and its analogs are allosteric A_2A_R inhibitors [17,18,69]. Subsequent studies revealed that the amiloride analog 5-(N,N-hexamethylene)-amiloride (HMA) is a potent allosteric A_2A_R inhibitor. The other amiloride analogs, benzamil, 5-(N-methyl-N-isobutyl)amiloride (MIBA), 5-(N-methyl- N-guanidinocarbonyl-methyl)amiloride (MCGMA), and phenamil, were found to be more effective allosteric inhibitors than amiloride at rat A_2A_Rs [17,70]. Moreover, amiloride and its analogues do not affect the dissociation rate of the agonist [^3^H]CGS21680 (3-{4-[2-({6-amino-9-[(2R,3R,4S,5S)-5-(ethylcarbamoyl)-3,4-dihydroxyoxolan-2-yl]-9H-purin-2-yl}amino) ethyl]phenyl}propanoic acid), but increase the dissociation rate of the antagonist [^3^H]ZM241385 (4-(2-{[7-amino-2-(furan-2-yl)[1,2,4]triazolo[1,5-a] [1,3,5] triazin-5-yl]amino}ethyl)phenol) from A_2A_Rs [71]. By contrast, sodium ions, for example, deteriorate the dissociation rate of the antagonist [^3^H]ZM241385 from A_2A_Rs in a dose-dependent manner [17]. It is important to note that other adenosine receptor agonists and antagonists are differentially affected by amilorides [70]. A new approach specifically targeting the sodium ion pocket, known as fragment-screening based on affinity mass spectrometry, led to the discovery of fragment Fg754 as a new A_2A_R NAM carrying a novel azetidine moiety and exhibiting inhibitory potency comparable to HMA. Subsequent simulations of the molecular dynamics, structure-activity relationship studies of the ligand, and nuclear magnetic resonance analyses in solution revealed the unique binding mode and antagonistic properties of Fg754, which is distinctly different from HMA [72]. In addition, cholesterol is reported to be a weak PAM of A_2A_Rs [73].

Identification of binding sites for allosteric modulators on A_2A_Rs based on the crystal structure of the receptor is critical for the development of new allosteric modulators. Two tightly linked residues, histidine residue number 278 (His^278^) in transmembrane domain 1 and glutamic acid^13^ in transmembrane domain 7 of the human A_2A_R, are reported to be the most crucial components for agonist recognition and play a partial role in the allosteric regulation by sodium ions [70,71,74,75,76,77]. Studies of the crystal structure of the antagonist-bound adenosine A_2A_R revealed that a highly conserved aspartate (Asp) residue in the second transmembrane domain is involved in sodium modulation of GPCRs [78]. Comparative studies of crystal structures in which a sodium ion bound in the allosteric site of human protease-activated receptor 1 [79], the β1-adrenergic receptor [80,81], the human δ-opioid receptor [82], and the human adenosine A_2A_R [78] show that sodium ions interact with the common residues Asp^2.50^ (superscript numbers refer to the Ballesteros and Weinstein residue numbering system [83]) serine^3.39^, tryptophan^6.48^, asparagine (Asn)^7.45^, and Asn^7.49^, either directly or through water-mediated hydrogen bonding [83]. Pre-crystal structure studies revealed that the positively charged sodium ion forms a permanent salt bridge with the negatively charged amino acid Asp^2.50^, suggesting that this residue represents the most conserved sodium ion binding site among GPCRs [84].

Subsequent studies on the crystal structure of the A_2A_R at 1.8 Å resolution provided sufficient resolution to confirm that Asp^2.50^ interacts directly with sodium ions via the salt bridge [78]. The crystal structures of agonist complexes for two variants in the first sodium coordination shell of the human A_2A_ adenosine receptor have also been reported [85]. A fluorine-19 nuclear magnetic resonance spectroscopy study suggested that A_2A_Rs have four distinct activation states; a partial agonist that favors the population of an active state (S_3_), an active state induced by full agonists (S_3′_), and two inactive states (S_1–2_); this study also demonstrated that sodium ions enhance the inactive states of A_2A_Rs [86]. In contrast, partial agonists and HMA induce active states, indicating that HMA competes with sodium ions for interaction with A_2A_Rs [84]. Moreover, all-atom simulations of molecular dynamics have shown that Fg754 can steadily enter the transmembrane domain core and form contacts with transmembrane helices 2, 3, 6, and 7, and extracellular loop 2. Particularly, the azetidine moiety of Fg754 may occupy the sodium ion-binding site by forming a salt bridge [72]. Another molecular dynamics simulation study described the allosteric effects of a mini-Gs protein on A_2A_Rs [87].

In conclusion, the effects of amiloride and its derivatives on A_2A_Rs are well studied. While the findings indicate that amiloride competes with sodium ions at the allosteric site of the A_2A_R, with Asp being the crucial amino acid, the allosteric binding site(s) of other small molecules selective for A_2A_Rs remain unknown.

## 6. Allosteric A_2A_R Modulators and Their Potential Clinical Application

Allosteric A_2A_R modulation could be a new target for drug discovery [88]. Allosteric modulators can selectively elicit a physiologic response where and when the orthosteric ligand is released, thereby reducing the risk of an adverse effect of A_2A_R activation. Moreover, the possibility of saturating allosteric effects offers greater potential for fine-tuning the physiologic response in a positive or negative direction. As allosteric modulators have no pharmacologic effect beyond the saturation dose, these molecules are associated with a lower risk for adverse effects than orthosteric ligands, giving them a potential therapeutic advantage over classical agonists and antagonists [18,89].

Some compounds act as allosteric A_2A_R modulators, such as sodium ions, amiloride, and potassium-sparing diuretics, that also modulate other GPCRs than A_2A_Rs [90]. For example, PD120918 is reported to enhance the activity of A_2A_R agonists in the rat striatum [91]. In contrast, thiadiazoles such as SCH-202676 alter the binding characteristics A_2A_R agonists and antagonists [92]. Some studies, however, suggest that thiadiazoles act as binding or oxidizing agents for SH groups rather than as allosteric modulators [92]. To date, only a relatively small number of selective allosteric A_2A_R modulators have been reported (Table 1) [93].

### 6.1. Allosteric A_2A_R Modulation Related to Inflammation

Adenosine is present in high concentrations in inflamed areas due to cell activation and breakdown [98,99,100]. The intracellular concentration of cAMP has a regulatory role in immune and inflammatory cells [101] and specifically, A_2A_Rs are responsible for the anti-inflammatory effects of adenosine [102,103]. The anti-inflammatory effects of A_2A_R agonists are well known. Their therapeutic benefit, however, is not a given due to the potential adverse effects of A_2A_R agonists following systemic administration [7].

AEA061, which has an undisclosed structure, promotes the anti-inflammatory effects of adenosine by allosterically enhancing the activity of endogenous adenosine at A_2A_Rs [96]. AEA061, which has no activity at a rat or human A_2A_Rs in the absence of adenosine, inhibits the production of cytokines such as interleukin-1α, macrophage inflammatory protein-1α, 1β, and 2, keratinocyte chemokine, RANTES (regulated upon activation, normal T cell expressed and presumably secreted), and tumor necrosis factor-α in monocytes and splenocytes in a mouse model of lipopolysaccharide-induced inflammation. Therefore, positive allosteric modulators of A_2A_Rs may represent a potential therapeutic approach to inflammation.

Inosine and inosine analog 6-S-[(4-nitrophenyl)methyl]-6-thioinosine (NBMPR) selectively and dose-dependently activate human A_2A_Rs. NBMPR and inosine inhibit the production of pro-inflammatory cytokines and chemokines in splenic monocytes of wild-type mice, but not A_2A_R knockout mice. The positive allosteric A_2A_R modulator AEA061 enhances inosine-mediated A_2A_R activation, inosine-mediated inhibition of pro-inflammatory cytokines, and chemokine production by splenic monocytes [97].

### 6.2. Allosteric A_2A_R Modulation Related to Sleep and Neurologic Disorders

**Figure 3:**
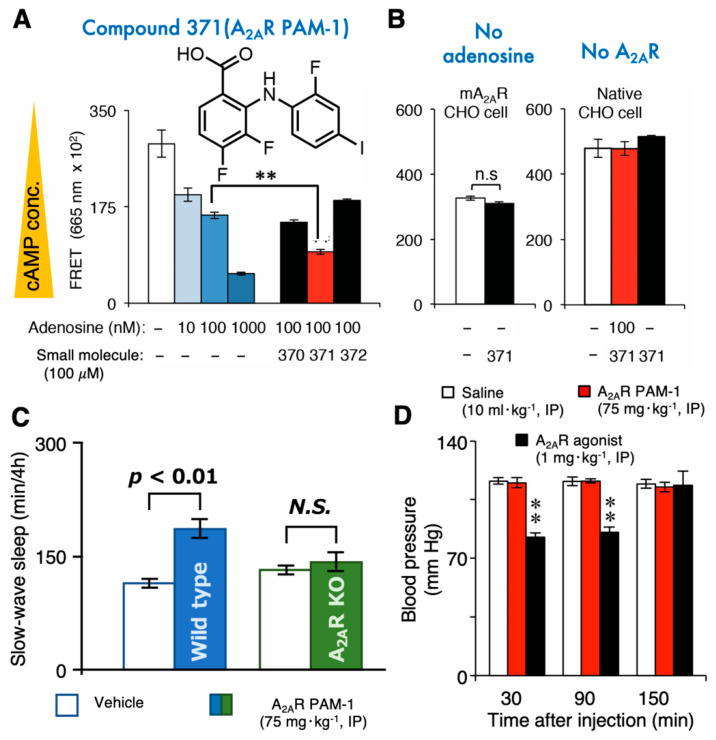
The A_2A_R positive allosteric modulator (PAM)-1 induces sleep without cardiovascular effects. (**A**,**B**) A_2A_R PAM-1 enhanced the activity of adenosine on A_2A_R-expressing Chinese Hamster Ovary (CHO) cells when cAMP was measured by a fluorescence energy transfer (FRET) immunoassay (**A**), whereas A_2A_R PAM-1 did not enhance cAMP production without adenosine or in native CHO cells without A_2A_R expression (**B**). (**C**) Intraperitoneal (IP) injection of A_2A_R PAM-1 increased slow-wave sleep in wild-type mice, but not in A_2A_R-knockout (KO) mice. (**D**) A_2A_R PAM-1 did not affect cardiovascular functions (e.g., blood pressure), unlike a classic A_2A_R agonist (CGS 21680) [94,95]. ** *p* < 0.01.

A_2A_Rs are also expressed in the CNS, with the highest levels in the ventral and dorsal striatum [104]. A_2A_Rs are present in the pre/postsynaptic compartment of neurons and microglia, oligodendrocytes, astrocytes, and capillary endothelial cells [12,105,106,107,108,109,110]. A growing number of reports illustrate that A_2A_Rs play a critical role in emotional and cognitive processes, motivation, and voluntary movements [111]. Moreover, A_2A_R-expressing neurons in the NAc regulate sleep [8,47,112]. Therefore, A_2A_R stimulation should be considered a potential treatment approach for insomnia. Insomnia is a sleep disorder that affects millions of people worldwide and frequently co-occurs with a wide range of psychiatric disorders [113,114,115] Although A_2A_R agonists have strong sleep-inducing effects [116,117,118,119], they also have adverse cardiovascular effects and thus cannot be used clinically to treat sleep disorders. Moreover, the development of adenosine analogs to treat CNS disorders, including insomnia, is hampered by the poor transport of these drugs across the blood–brain barrier. In mice, a small blood–brain barrier-permeable monocarboxylate (3,4-difluoro-2-((2-fluoro-4-iodophenyl)amino) benzoic acid, denoted as A_2A_R PAM-1, was recently found to induce sleep by enhancing A_2A_R signaling in the brain (Figure 3) but, surprisingly, did not exhibit the typical unwanted cardiovascular and body temperature effects of A_2A_R agonists [94,95]. More specifically, A_2A_R PAM-1 dose-dependently enhanced A_2A_R signaling in A_2A_R-expressing Chinese hamster ovary (CHO) cells but not in CHO cells lacking A_2A_R expression or in the absence of adenosine (Figure 3). The A_2A_R PAM-1 did not alter the activity of the A_2A_R agonist CGS 21680 [120]. Intracerebroventricular infusion and intraperitoneal injection of A_2A_R PAM-1 induced prolonged slow-wave sleep, but not rapid-eye-movement sleep, in wild-type mice, but not A_2A_R knockout mice. Further testing revealed that A_2A_R PAM-1, unlike A_2A_R agonists, had no effects on blood pressure, cardiac function, or body temperature, suggesting that adenosine or A_2A_R expression levels in the cardiovascular system are insufficient to elicit an A_2A_R PAM-1 response under normal physiologic conditions. Therefore, molecules that allosterically enhance A_2A_R signaling may be developed to help people with insomnia fall asleep more easily. Moreover, A_1_Rs play a crucial role in the resolution of sleep need by modulating slow-wave activity, a slow, oscillatory neocortical activity that intensifies in correlation with wake duration and declines during sleep [121]. Slow-wave activity is widely used as a marker of mammalian sleep homeostasis and is necessary for sleep function. Therefore, dual allosteric A_1_R/A_2A_R modulators may be useful for improving not only the maintenance of sleep but also its function.

A_2A_Rs have roles in neurodegenerative, neurodevelopmental, and psychiatric diseases. The potential therapeutic use of A_2A_R agonists and antagonists for specific conditions such as Niemann Pick disease, schizophrenia, autism-spectrum disorders, depression, anxiety, Alzheimer’s disease, attention-deficit hyperactivity disorder, PD, and fragile X syndrome is comprehensively discussed in the literature [122]. Allosteric A_2A_R modulators may provide alternative therapeutic options for neurologic disorders to circumvent the complexity of central and peripheral adenosine signaling. For example, dopamine-replacement therapy in PD is potentiated by blocking A_2A_Rs due to the adenosine-dopamine antagonism in the striatum [123]. Decade-long preclinical studies of A_2A_R antagonists in PD models led to clinical trials of the A_2A_R antagonist istradefylline, which confirmed its clinically significant motor benefit in advanced PD patients and resulted in the approval of istradefylline for the treatment of PD patients in Japan and the US. The complexity of adenosine signaling contributed at least partially to the debilitating side effects and suspension of the clinical phase III trial of the A_2A_R antagonist tozadenant for PD, which resulted in the death of five patients due to inflammatory complications. Thus, there is also a critical need to develop safer and more effective means of suppressing A_2A_R signaling; for example, by negative allosteric modulation. Whereas the most potent PD medication is levodopa (l-3,4-dihydroxyphenylalanine), clinicians try to limit levodopa doses to the extent possible to avoid various adverse effects occurring with chronic use, such as dyskinesia and dopamine dysregulation. A_2A_R PAM, when administered concomitantly with levodopa, may mitigate some of these side effects, but strong evidence is currently lacking.

In addition, positive allosteric modulators of A_2A_Rs may also alleviate various symptoms in neuropsychologic disorders. For example, psychotic symptoms such as delusions are caused by impaired discrimination of environmental stimuli. Recent evidence shows that D_2_Rs mediate discrimination learning in the NAc, but A_2A_Rs expressed together with D_2_Rs in the NAc are required for discrimination learning. While normal mice can discriminate between reward-predictive and non-reward-predictive tones several days after generalized reward conditioning (when any tone is reward-predictive), mice in which A_2A_Rs are blocked in the NAc do not show this ability [124]. In addition, hypofunction of NMDA-type glutamate receptors is thought to be involved in schizophrenia, as NMDA receptor antagonists such as phencyclidine and dizocilpine (MK-801) cause psychotic and cognitive disorders in humans and animals [125]. Deleting A_2A_Rs in NAc astrocytes leads to motor and memory impairments relevant to schizophrenia, namely exacerbation of the MK-801-induced psychomotor response and impaired working memory [126]. Thus, the enhancement of A_2A_R signaling may be helpful to treat sleep disorders as well as schizophrenia and other psychotic disorders by overcoming dopaminergic hyperactivity or glutamatergic hypoactivity.

## 7. Concluding Remarks

Here, we discussed recent developments regarding allosteric A_2A_R modulation. Although numerous allosteric modulators of A_2A_Rs have been identified, the physiologic functions of only a few of them have been established. The sleep-promoting effects and inflammatory process-modulating roles of allosteric A_2A_R modulators open the doors for the potential therapeutic use of these molecules for treating diseases. Allosteric modulators exert their effects only where and when the orthosteric ligand is released, conferring a potential therapeutic advantage over classical antagonists and agonist molecules. Thus, allosteric A_2A_R modulation could provide patients with an effective and safe treatment for various diseases.

Finally, A_2A_Rs form heterodimer structures with other receptors such as D_2_Rs and mGluR5 in the CNS. Receptor heterodimers may be an applicable target for developing A_2A_R PAMs with high specificity for the heterodimer and thus limited adverse effects [38,127,128,129,130,131].

## Figures and Tables

**Table 1:** Allosteric A_2A_R modulators and their functions.

Name	Type	Pharmacology	Structure	Physiologic Effects
3,4-Difluoro-2-((2-fluoro-4-iodophenyl)amino)benzoic acid	Allosteric enhancer/modulator	Enhanced adenosine signaling at mouse A_2A_Rs.	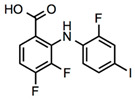	Induced slow wave sleep without affecting cardiovascular function or body temperature in wild-type male mice [94,95].
AEA061	Allosteric enhancer/modulator	Enhanced adenosine and inosine signaling and increased effect of the A_2A_R agonist CGS 21680.	Not disclosed	Inhibited the production of tumor necrosis factor-α, macrophage inflammatory protein-1α, 1β, and 2, interleukin-1α, keratinocyte chemokine, and RANTES (regulated upon activation, normal T cell expressed and presumably secreted) in macrophages and splenocytes, reduced circulating plasma tumor necrosis factor-α and monocyte chemoattractant protein-1 levels, and increased plasma interleukin-10 during lipopolysaccharide-induced endotoxemia [96,97].
N-(3-Benzyl-5-phenyl-3H-[1,2,3]triazolo[4,5-d]- pyrimidin-7yl-)-(4-aminophenyl)-amine	Allosteric modulator	Inhibited the binding of antagonists and agonists at the A_2A_R orthosteric site [93].	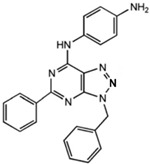	Unknown
N^6^-[(4-Nitro)-phenyl]-9-benzyl-2-phenyladenine	Allosteric modulator	Inhibited the binding of antagonists and agonists at the A_2A_R orthosteric site [93].	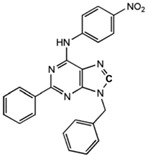	Unknown
N^6^-[(4-Amino)-phenyl]-9-benzyl-2-phenyladenine	Allosteric modulator	Inhibited the binding of antagonists and agonists at the A_2A_R orthosteric site [93].	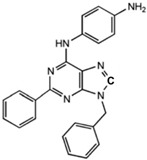	Unknown
1-[4-(3-Benzyl-5-phenyl-3H-[1,2,3]triazolo[4,5-d]-pyrimidin-7-ylamino)-phenyl]-3-(4-fluorophenyl)-urea	Allosteric modulator	Modulated the binding of antagonist and agonist at the A_2A_R orthosteric site [93].	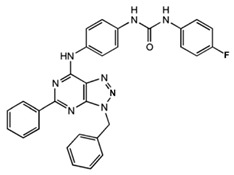	Unknown
1-[4-(3-Benzyl-5-phenyl-3H-[1,2,3]triazolo[4,5-d]-pyrimidin-7-ylamino)-phenyl]-3-(4-trifluoromethylphenyl)- urea	Allosteric modulator	Modulated the binding of antagonist and agonist at the A_2A_R orthosteric site [93].	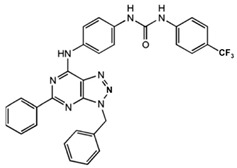	Unknown
1-[4-(9-Benzyl-2-phenyl-9H-purin-6-ylamino)- phenyl]-3-(4-methoxyphenyl-urea	Allosteric modulator	Modulated the binding of antagonist and agonist at the A_2A_R orthosteric site [93].	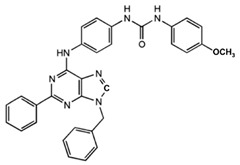	Unknown
Amiloride	Allosteric modulator	Increased the dissociation rate of the antagonist ZM-241,385 at rat A_2A_Rs [18,71].	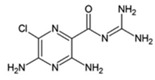	Unknown
Benzamil	Allosteric modulator	Increased the dissociation rate of the antagonist ZM-241,385 at rat A_2A_Rs [71].	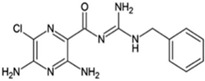	Unknown
HMA; 5-(N,N-hexamethylene)amiloride	Allosteric modulator	Increased the dissociation rate of the antagonist ZM-241,385 at rat A_2A_Rs [71].	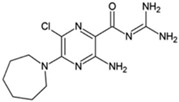	Unknown
MGCMA; 5-(N-methyl-N-guanidinocarbonyl-methyl)amiloride	Allosteric modulator	Increased the dissociation rate of the antagonist ZM-241,385 at rat A_2A_Rs [71].	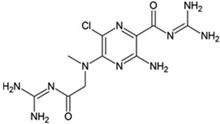	Unknown
MIBA; 5-(N-methyl-N-isobutyl)amiloride	Allosteric modulator	Increased the dissociation rate of the antagonist ZM-241,385 at rat A_2A_Rs [71].	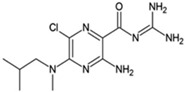	Unknown
Phenamil	Allosteric modulator	Increased the dissociation rate of the antagonist ZM-241,385 at rat A_2A_Rs [71].	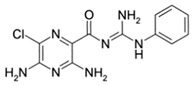	Unknown
Sodium Ion	Allosteric modulator	Positively modulated A_2A_Rs [71].	Na^+^	Unknown
PD120918 {4-methyl-7-[(methyl- amino)carbonyl]oxy}-2H-1-benzopyran-2-one}	Allosteric modulator	Enhanced agonist radioligand binding to rat striatal A_2A_Rs without functional enhancement [18,91].	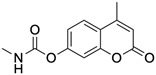	Unknown
Fg754	Allosteric modulator	Increased the dissociation rate of the agonist CGS21680 at A_2A_Rs expressing HEK-293 cells [72].	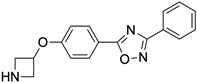	Unknown
Cholesterol	Allosteric modulator	Decreased the dissociation rate of the agonist NECA at A_2A_Rs-embedded nanodiscs [73].	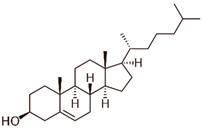	Unknown
